# The Gut Microbiome–Brain Crosstalk in Neurodegenerative Diseases

**DOI:** 10.3390/biomedicines10071486

**Published:** 2022-06-23

**Authors:** Laura Ghezzi, Claudia Cantoni, Emanuela Rotondo, Daniela Galimberti

**Affiliations:** 1 Department of Neurology, School of Medicine, Washington University, St. Louis, MO 63110, USA; claudiacantoni@wustl.edu; 2 Neurodegenerative Diseases Unit, Fondazione IRCCS Ca’ Granda Ospedale Maggiore Policlinico, 20100 Milan, Italy; emanuela.rotondo@gmail.com (E.R.); daniela.galimberti@unimi.it (D.G.); 3 Department of Biomedical, Surgical and Dental Sciences, University of Milan, Centro Dino Ferrari, 20100 Milan, Italy

**Keywords:** gut microbiome, gut–brain axis, neurodegenerative diseases

## Abstract

The gut–brain axis (GBA) is a complex interactive network linking the gut to the brain. It involves the bidirectional communication between the gastrointestinal and the central nervous system, mediated by endocrinological, immunological, and neural signals. Perturbations of the GBA have been reported in many neurodegenerative diseases, suggesting a possible role in disease pathogenesis, making it a potential therapeutic target. The gut microbiome is a pivotal component of the GBA, and alterations in its composition have been linked to GBA dysfunction and CNS inflammation and degeneration. The gut microbiome might influence the homeostasis of the central nervous system homeostasis through the modulation of the immune system and, more directly, the production of molecules and metabolites. Small clinical and preclinical trials, in which microbial composition was manipulated using dietary changes, fecal microbiome transplantation, and probiotic supplements, have provided promising outcomes. However, results are not always consistent, and large-scale randomized control trials are lacking. Here, we give an overview of how the gut microbiome influences the GBA and could contribute to disease pathogenesis in neurodegenerative diseases.

## 1. Introduction

The gut–brain axis (GBA) refers to a complex network of bidirectional interactions between the gut microbiome and the central nervous system (CNS). The GBA involves multiple biological systems and is crucial in maintaining the overall body homeostasis [1]. Signals travel from the gut to the CNS and vice versa, either directly through the autonomic nervous system or indirectly through metabolites and chemical transmitters [1,2]. Both of these interactions can modulate and be influenced by the gut microbiome composition. The GBA has recently attracted interest due to its emerging role in mediating health and disease and potential use as a therapeutic target. The gut microbiome impacts many aspects of brain development and function, including microglia and astrocyte maturation and polarization, blood–brain barrier (BBB) formation and permeability, neurogenesis, and myelination [3,4,5,6,7,8,9]. GBA disruption may participate in the pathophysiology of several brain disorders, including neurodegenerative diseases [5,10,11,12]. However, controversy exists surrounding the extent and the exact mechanisms through which an altered gut microbiome may influence the development of CNS inflammation and degeneration. This review summarizes the current literature exploring the GBA’s role in neurodegenerative diseases, focusing on current and potential therapeutic strategies targeting the GBA.

## 2. The Gut Microbiome in CNS Homeostasis

**Figure 1:**
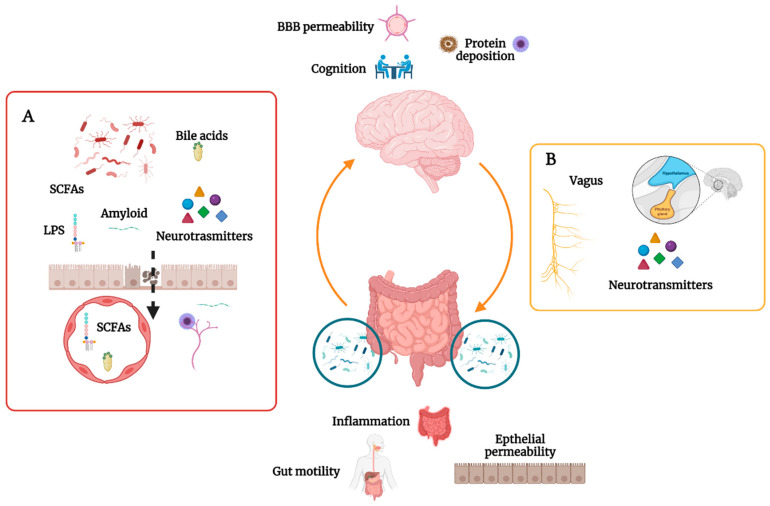
The gut microbiome influences the gut–brain axis through the production of SCFAs, amyloid proteins, LPS, bile acids, and neurotransmitters. SCFAs and bile acids can enter the circulation and have been demonstrated to have an important effect on maintaining BBB homeostasis. Amyloid proteins and LPS can increase local inflammation, promoting further local and systemic protein deposition (**A**). On the other hand, the CNS can control epithelial permeability, gut motility, and inflammation through the autonomic nervous system and the hypothalamus–pituitary axis (**B**). Created with *Biorender.Com*.

The gut microbiome is constituted by a huge community of bacteria, archaea, fungi, and viruses [13], with a great deal of variability among individuals. Unfortunately, there is no agreement on which populations should prevail in a healthy adult microbiome. Cross-sectional studies have shown that gut microbiome composition in individuals with various neurological diseases is different compared to healthy controls [1] (Table 1). Moreover, preclinical studies in animal disease models have confirmed that the gut microbiome obtained from patients with neurological diseases can precipitate brain pathology and behavioral changes in mice [14]. Gut microorganisms can influence CNS functions by producing metabolites, neuroactive molecules, and hormones [15] (Figure 1).

Short-chain fatty acids (SCFAs) are the main products of anaerobic fermentation of indigestible polysaccharides such as dietary fibers and resistant starch by the microbiome in the large intestine [40]. They comprise mainly acetate, propionate, and butyrate, and their quantity varies depending on diet and the gut microbiome composition [41]. SCFAs improve gut health by maintaining the intestinal barrier, mucus production, and immunoregulation [42]. Although the exact mechanisms of action of SCFAs remain unknown, it is known that SCFAs bind to G protein-coupled receptors (GPCRs). The best-studied SCFAs receptors are G-protein coupled receptors 41 and 42 (GPR42 and GRP41), also known as free-fatty acid receptors 2 and 3 (FFAR2 and FFAR3), which are expressed on a wide variety of cells and whose activation determines a different effect based on the cell type harboring them [43]. Besides exerting local effects, SCFAs can cross the BBB and play an important role in the microbiome–brain crosstalk. In animal models, the brain uptake of SCFAs has been demonstrated following the injection of 14C-SCFAs into the carotid artery. In humans, detectable levels of SCFAs in cerebrospinal fluids (CSF) have been reported [44]. SCFAs seem to contribute to BBB integrity. Supporting this notion, germ-free (GF) mice show reduced expression of tight junction proteins such as claudin and occludin, leading to increased permeability of the BBB from intrauterine life to adulthood [4]. Recolonization of these mice with SCFA-producing bacterial strains rescues the phenotype [4]. Glial cells, especially microglia, play a pivotal role in sculpting neuronal networks and shaping circuits. Normal microglial functions are fundamental for the elimination of excess or unnecessary synaptic connection during CNS development [45]. The microbiome influences microglial maturation and function, as demonstrated by the immature microglia phenotype described in GF mice [46]. It is interesting to note that SCFA supplementation can reverse microglia defects [46]. Although the mechanisms involved in controlling the maturation and function of microglia by SCFAs remain unknown, the activation of FFAR2 seems to be involved, since FFAR2-deficient mice displayed the same microglia defect as GF mice [47].

Secondary bile acids are another category of microbial-derived metabolites that has recently attracted a lot of attention in the context of the GBA. Bile acids are endogenous molecules synthesized by the liver from cholesterol and further metabolized by the gut microbiome. Primary bile acids are secreted in the intestine where they are either reabsorbed by the ileum mucosa or deconjugated by the gut microbiome [48]. It is known that microbial deconjugation prevents bile acid reuptake, but studies on GF mice showed how the gut microbiome is also fundamental to regulating primary bile acid reabsorption at the ileum level [49]. In addition, both conjugated and unconjugated bile acids have been demonstrated to cross the BBB, and bile acid receptors are present in the CNS [50]. Alterations in bacterial-associated bile acids have been reported in human studies and mouse models of PD, AD, and other neurological diseases [51,52]. However, the exact function of bile acids in the GBA crosstalk has not been elucidated yet.

Lipopolysaccharide (LPS) is a large molecule present in the outer membrane of Gram-negative bacteria. In the CNS, released LPS is recognized by toll-like receptors 4 (TLR4), primarily expressed by microglia with subsequent production of proinflammatory cytokine and proliferation [53]. The opposite effect can be exerted by polysaccharide A, secreted by *B. fragilis* and recognized by toll-like receptors 2 (TLR2), whose activation induces a protective CNS immunoregulatory response [54].

Gut hormones are also important to consider in gut–brain signaling. Obesity has been correlated to mood disorders, and several gut hormones (i.e., cholecystokinin, ghrelin, and serotonin) have been linked to anxiety and depression [55].

Gut microbes can either modulate or directly synthesize neuroactive substances such as dopamine, noradrenaline, acetylcholine, histamine, melatonin, and gamma-aminobutyric acid (GABA) [15]. GABA-producing pathways are expressed by *Bacteroides, Parabacteroides*, and *Escherichia* species [56]. Evidence of their influence on CNS synaptic transmission, through the gut–brain axis, is derived from studies on subjects with major depressive disorder (MDD), a disease strongly associated with GABA dysregulation. In MDD patients, the relative abundance of fecal *Bacteroides* is inversely correlated with the functional connectivity between the dorsolateral prefrontal cortex and the default mode network [56]. Furthermore, modulation of the gut microbiome composition has been demonstrated to influence both circulating and central levels of GABA [57,58]. Treatment of mice with the probiotic strain *Lactobacillus rhamnosus* (JB-1) showed a reduction of stress and depression-like behavior (assessed by the forced-swim test), associated with the modulation of GABA receptor expression in the prefrontal cortex, the amygdala, and the hippocampus [57]. In another study, the administration of *Lactobacillus* and *Bifidobacterium* increased the levels of GABA inside the CNS and ameliorated memory performances in mice [59]. Gut bacteria and enteroendocrine cells (EECs) are also important in the production of serotonin (5-HT), and even if the majority of 5-HT is produced in the gut and cannot bypass the BBB, GF mice have decreased concentrations of 5-HT and tryptophan in the CNS, suggesting a modulating role of the gut microbiome on central serotonin levels [60]. The production of 5-HT by EECs is influenced by the levels of secondary bile acids [61].

## 3. The Gut Microbiome and Cognition

Microbiome composition is age-sensitive, and humans show marked differences in microbial profiles during infancy, adolescence, adulthood, and aging [62,63,64]. Many factors shape the developing microbiome: Genetics [65], stress [66], mode of birth [67], diet [68], medication [69], and the environment [70]. Before birth, bacteria present in the placenta, amniotic cavity, umbilical cord, and meconium start to shape the characteristics of the future microbiome [71].

After birth, and during the first years of life, the gut microbiome of infants experiences significant changes, mainly influenced by feeding patterns (breast vs. artificial milk and, later, solid food). However, diet remains the main determinant of the gut microbiome composition in adults.

The gut microbiome has an intrinsic role in aging-related cognitive impairment. The dysbiotic status, characteristic of the aging microbiome, influences cognition through multiple pathways. The prevalence of bacteria considered proinflammatory, at the expense of more immunoregulatory microbial populations, can promote the release of pro-inflammatory cytokines and bacterial toxins, inhibit the transmission of the regulatory neural signal via the vagus nerve, and suppress the production and release of microbial metabolites and hormones [72,73].

From a neuropsychological point of view, several studies found a correlation between the gut microbiome composition and performance on cognitive tests (motor speed, attention, memory). For example, the relative abundance of *Actinobacteria* phylum has been linked to better performance in tests of attention, working memory, and paired-associate learning tasks [74]. In a mouse model, the administration of two *Bifidobacterium* strains improved memory and learning [75]. In humans, the administration of B. longum for 4 weeks was associated with reduced stress and improved visuospatial memory performance [76]. However, in another study, administration of the probiotic *Lactobacillus casei* Shirota to a cohort of healthy middle-aged subjects determined an improvement in mood but a parallel slight decrease in memory performance in neurocognitive tests [77]. Therefore, it is currently far from clear whether supplementation with psychobiotics, i.e., probiotics with an effect on the CNS, can exert any consistent effects on cognition in humans.

Furthermore, many of the benefits observed in learning and memory after the administration of probiotics occurred alongside reductions in biomarkers of stress (glucocorticoids) or inflammation (proinflammatory cytokines) [76]. Both glucocorticoids and proinflammatory cytokines impair cognitive performance under numerous conditions [78]. As stated above, dysbiosis is associated with impaired gut barrier function that allows bacteria or bacterial products to infiltrate into systemic circulation [79]. The resulting inflammatory states have been associated with behaviors such as social isolation, depression, apathy, and attentional impairments.

## 4. The Gut Microbiome and Parkinson’s Disease

Parkinson’s disease (PD) is a progressive neurodegenerative disease characterized by the loss of dopaminergic neurons in the substantia nigra and striatum, with abnormal accumulation of α-synuclein in the brain. The main symptoms of PD are resting tremors, stiffness, bradykinesia, and postural instability [80]. With disease progression, cognitive decline might ensue [81]. In addition, non-motor symptoms such as behavioral changes, sleep disorders, and gastrointestinal and autonomic dysfunction may precede the motor symptoms [82]. More than 80% of patients with PD experience gastrointestinal symptoms [83]. As in other neurodegenerative diseases, PD has been associated with inflammation, specifically the inflammatory state resulting from the senescence of the immune system, defined as inflammaging [84]. In 2003, Braak and collaborators introduced the hypothesis that PD originates in the gut [85] and dysbiosis and gut inflammation are now considered important contributors to the disease pathogenesis [86,87]. Inflammatory responses have been described in the colonic tissues of animal models of the disease, including the elevation of proinflammatory cytokines and chemokines such as TNF-α, IL-1b, and leukocyte infiltration and activation [88,89]. In people with PD, serum levels of calprotectin, a marker of intestinal inflammation, were reported elevated compared to healthy controls (HCs) and correlated with monocyte count in the peripheral blood [90]. In another study, calprotectin, alpha-1-antitrypsin, and zonulin were also increased in people with PD [91]. Studies focused on gut microbiome changes in people with PD described a significant increase in the proinflammatory bacteria *Ralstonia, Akkermansia, Oscillospira*, and *Bacteriodes* [26]. *Bacteroides* and *Verrucomicrobiaceae* abundance have also been associated with plasma TNF-α and IFN-γ levels, respectively [33]. Another consistently reported alteration in PD patients is a decrease in *Roseburia* abundance [12]. *Roseburia* can enhance intestinal barrier function and reduce intestinal inflammation by upregulating antimicrobial peptide genes and toll-like receptor (TLR)-related genes, such as *TLR5*, and downregulating the NF-kB pathway [92]. Consistently, sigmoid mucosa biopsies obtained from patients with PD showed an increase in the expression of TLR4 mRNA compared to HCs [93]. In the rotenone mouse model, loss of the *TLR4* gene significantly improved intestinal barrier integrity and reduced intestinal and CNS inflammation, α-synuclein aggregation, and dopaminergic cell loss in the substantia nigra, thus alleviating the impairment of motor function [93].

Several animal studies have reported the spread of α-synuclein pathology from the gut to the brain, and pathological changes in the CNS can be observed after the injection of α-synuclein into the intestinal wall [94,95,96]. However, the exact route through which pathological deposits of α-synuclein may spread to the brain remains vastly hypothetical. According to Braak’s original hypothesis, environmental factors may contribute, triggering the pathological process via the olfactory bulb or the intestinal nerve plexus [97]. Supporting this theory, vagotomy can prevent the transmission of pathological alpha-synuclein to the CNS in animal models [95].

## 5. The Gut Microbiome and Alzheimer’s Disease

AD is the most common cause of cognitive decline worldwide [98]. It is characterized by the deposition of Amyloid beta (Aβ) plaques and hyperphosphorylated tau protein tangles, leading to neuroinflammation, synaptic dysfunction and, ultimately, neuronal loss [98]. As with PD, AD pathogenesis has also been linked to GBA dysfunction and increased intestinal inflammation [99]. The evidence of the possible role of the gut microbiome in AD pathogenesis came from the mouse model of the disease. GF APPPS1 mice showed a reduction in Aβ pathology compared to specific pathogen-free (SPF) mice [100]. Moreover, some recent studies have reported an altered gut microbiome composition in people with AD compared to HCs [10,17,18,19]. Aging itself impacts the gut microbiome composition, favoring proinflammatory bacteria, such as *Bacillus fragilis, Bacteroides fragilis*, and *Faecalibacterium prausnitzii*, to the detriment of more immune-regulatory bacteria [101]. Indeed, in patients with evidence of amyloid deposition, an increase in the proinflammatory taxa *Escherichia* and *Shigella* was associated with an increase in peripheral inflammatory markers such as interleukin-1β, NLR Family Pyrin Domain Containing 3, and C-X-C Motif Chemokine Ligand 2 (IL-1β, NLRP3, and CXCL2) [16,17,18]. The alterations most consistently reported by other studies include a decrease in *Firmicutes* and *Bifidobacteria*, together with an increase in *Proteobacteria* and *Enterobacteria* [16,17,18]. However, the results regarding *Bacteroidaceae* abundance seem to be less reproducible, with studies reporting either a decrease [22] or an increase [21], variably associated with alteration in *Actinobacteria* or *Prevotella*. The lack of consistency can be explained by the different geographical origins of the participants and the difference in comorbidities [102]. Interestingly, a pronounced difference in *Enterobacteriaceae* abundance between AD and MCI has been reported, suggesting the changes in gut microbiome composition might be gradual during disease progression [19].

As far as gut microbial products are concerned, an alteration in the gut’s production of SCFAs, including butyrate, propionate, and acetate, has been repeatedly reported in patients with AD [101]. A decrease in SCFAs has been associated with increased epithelial leakage and bacterial translocation, with a consequent increase in circulating Gram-negative bacteria and LPS [103], microglia activation, and Aβ deposition in the CNS [104,105]. Moreover, a lower abundance of the butyrate-producing genus *Butyrivibrio* has been linked to a reduction in the intestinal expression of the transporter P-glycoprotein in AD patients [18]. Intestinal P-glycoprotein has been demonstrated to be essential for maintaining gut homeostasis and controlling intestinal inflammation [106]. Butyrate seems to have a protective effect against neuroinflammation in animal models of disease [107]. However, studies on the effect of SCFA supplementation reported contrasting results, with an even higher Aβ burden after treatment with butyrate in one study [104]. Besides acting locally, gut-derived metabolites can penetrate the CNS [108]. Elevated levels of trimethylamine N-oxide have been reported in people with AD and MCI compared to HCs and are correlated with Aβ and p-tau levels [109].

Lastly, as outlined in the previous paragraph, strains of bacteria derived from the gut microbiome can produce amyloids and favor Aβ peptide aggregation [101]. The proteins curli, TasA, CsgA, FapC, and phenol soluble modulins produced by *E. coli, B. subtilis, S. typhymurium, E. fluorescens*, and *S. aureus*, respectively, are only some of the bacteria-derived amyloids with the ability to promote the formation of Aβ oligomers and fibrils in vitro [110]. Bacterial-derived amyloids can act in concert with other bacterial-derived products, such as LPS, to trigger inflammation and increase Aβ deposition in the CNS, as it has been extensively demonstrated in animal models of AD [111,112,113].

## 6. The Gut Microbiome and Amyotrophic Lateral Sclerosis/Frontotemporal Dementia

Compared to other neurodegenerative disorders, little evidence supports the implication of the gut microbiome in amyotrophic lateral sclerosis (ALS) and frontotemporal lobar dementia (FTD).

ALS is a fatal neurodegenerative disease characterized by the progressive loss of motor neurons [114]. One of the most common mutations associated with familial ALS is found in the superoxide dismutase 1 gene (SOD1) [114]. By looking at humanized SOD1 mutated mice (G93A), researchers found that these mice had decreased intestinal integrity, increased intestinal permeability, and abnormal Paneth cells, an important cluster of cells implicated in autophagy host–pathogen interactions. Moreover, G93A mice showed an atypical intestinal microbiome, meager in SCFA-producing bacteria such as *Butyrivibrio fibrisolvens* [115]. Interestingly, butyrate supplementation successfully restored intestinal eubiosis and prolonged G93A mouse life spans [116].

Besides SCFA, nicotinamide supplementation also helps the improvement of ALS symptoms in the mouse model. In particular, the lack of *Akkermansia muciniphila*, a bacterium that produces a high quantity of nicotinamide, has been associated with a worse clinical course, and nicotinamide supplementation produced a partial improvement. Notably, changes in the gut microbiome composition were reported in the mouse model before clinical onset, opening the possibility of using the gut microbiome as an early biomarker of disease. Unfortunately, studies on people with ALS led to inconsistent results, possibly due to differences in sample size and patient characteristics [35,36]. However, recent studies seem to agree that ALS patients show a noticeable change in the gut microbial structure when compared to healthy controls, consisting of the increased abundance of the *Bacteroidetes* phylum, together with a decrease in *Firmicutes* [37], *Roseburia intestinalis* and *Eubacterium rectale*, two dominant butyrate-producing species [38].

Frontotemporal dementia (FTD) is a term referring to a wide variety of syndromes, including the behavioral variant frontotemporal dementia and the non-fluent and semantic variants of primary progressive aphasia, each of which can also be accompanied by ALS [117]. Studies on the GBA in people with FTD are scarce and limited to the animal model of the disease. FTD is a multifactorial disease with a solid genetic contribution and a variable degree of environmental influence. The three most common genes involved in the development of the disease are Chromosome 9 Open Reading Frame (*C9ORF*)*72*, Microtubule-Associated Protein Tau (*MAPT*), and Progranulin (*GRN*). The expansion of *C9ORF72* is considered the most common genetic cause of FTD and ALS [118]. As with *GRN* mutations, another less common genetic cause of FTD, *C9ORF72* expansion, is always associated with TAR DNA binging Protein (TDP)-43 pathology. *MAPT* mutations are invariably associated with Tau pathology [117].

While the loss of *C9ORF72* function in humans is associated with neurodegeneration, C9ORF72 reduction or complete deletion in knockout mice (*C9orf72^−/−^*) does not trigger ALS or FTD-like disease [119]. *C9orf72^−/−^* present an inflammatory phenotype characterized by cytokine storm, splenomegaly, and neuroinflammation [120]. Remarkably, antibiotic treatment or fecal transplantation from an anti-inflammatory environment-associated mouse phenotype was able to rescue the phenotype [120].

Finally, microbiome studies on *Drosophila* flies carrying the transgenic mutant human FTDP-17-associated tau showed reduced gut motility and subsequently increased gut bacterial load in aged tau transgenic compared to control flies [121].

## 7. The Gut Microbiome and Other Forms of Dementia

Alterations in the GBA have been reported in Lewy body dementia (LBD), Huntington’s disease (HD), and Creutzfeldt–Jakob disease (CJD). LBD is associated with impaired control of gastrointestinal and cardiac functions that might be linked to the loss of cholinergic dorsal vagal nucleus (DMV) neurons. The degree of DMV cell loss has been found to be similar in LBD patients with or without gastrointestinal symptoms [122].

HD is an inherited neurodegenerative disease that causes progressive motor decline, cognitive dysfunction, and neuropsychiatric symptoms. In addition to the neurological decline and similar to LBD, HD is associated with gastrointestinal disturbances, nutrient deficiencies, gastritis, and weight loss [123]. As reported in PD, AD, and ALS, one study found lower alpha and beta diversity, indicating less healthy baseline richness and altered microbial gut composition in HD patients than HCs [124].

Prion diseases, also known as transmissible spongiform encephalopathies (TSEs), are a group of fatal neurodegenerative diseases affecting humans and animals. Studies from three decades ago showed how germ-free mice infected with prions have increased survival compared to conventional mice, suggesting a role of microbes in enhancing the disease [125]. Moreover, the prion disease mouse microbiome is significantly different from non-infected mice, alongside SCFA and bile acid production [126].

## 8. Therapeutic Approaches

### 8.1. Fecal Microbiome Transplantation

Fecal microbiome transplantation (FMT) is a procedure where a solution of fecal material from a donor is transferred, through colonoscopy, nasogastric tubes, or oral pills, into the intestinal tract of a recipient, aimed at directly changing the gut microbiome composition [127]. FMT has shown promising results in C. difficile infection [128] and could potentially be applied to all the diseases associated with gut microbiome dysbiosis, including neurodegenerative diseases. The most robust results on the effects of FMT on neurodegenerative diseases have been obtained from studies on animal models [101]. FMT from mouse models of AD to GF or antibiotic-treated specific-pathogen-free (SPF) recipient mice resulted in impaired neurogenesis, memory impairment, increased levels of proinflammatory cytokines, and Aβ plaque deposition [129,130]. Furthermore, when FMT was carried out from people with AD to GF mice, an accelerated cognitive decline was observed [131]. On the contrary, ADLPAPT and APPswe/PS1dE9 Tg mice receiving FMT from WT mice showed less cognitive decline, lower Aβ burden, and lower levels of systemic inflammation [132,133,134]. In humans, only two single-case report studies have been published so far. Both showed improvement in cognitive function in people with AD receiving FMT from HCs [135,136].

FMT from animal models of PD into healthy mice could induce synaptic loss and motor dysfunction. On the contrary, FMT from healthy mice to MTPT-treated mice showed a neuroprotective effect and suppressed the activation of TLR4/TNF-α pathway in the gut and the brain [137]. Moreover, α-synuclein-overexpressing mice colonized with the gut microbiome of people with PD showed exacerbated motor dysfunctions [138]. Based on the few case reports published, FMT is reported to be safe and effective at reducing constipation and temporally relieving motor symptoms in people with PD [139,140,141]. Despite promising results in preliminary studies on animal models and human subjects, caution is needed when drawing conclusions, and future randomized clinical trials are needed to clarify the role of FMT in neurodegenerative diseases. FMT is associated with minor adverse events and side effects, including diarrhea, abdominal pain, fever, and flatulence [139,140,141]. However, the exact mechanisms behind its effects on the CNS are still poorly understood and the effect of a single treatment is only temporary. Significant challenges include the timing of transplantation, the high costs of sample preparation and preservation, and the difficulties in defining the criteria to select fecal donors [12]. 

### 8.2. Probiotics

Probiotics are defined as non-viable food components that confer health benefits to the host and are associated with modulating the microbiome [12]. Studies on human and animal models have shown that probiotics can help maintain intestinal homeostasis, by stabilizing the epithelial barrier, increasing the production of SCFAs, modulating the mucosal immune system towards a more immunoregulatory response, and inhibiting the production of proinflammatory cytokines [101]. In animal models of AD, the administration of a probiotic mixture showed enhancement of cognitive functions, modulation of the gut microbiome composition, and reduction of oxidative stress and CNS inflammation [142,143,144]. In addition, the administration of a probiotic mixture of Lactobacillus and Bifidobacterium has proven capable of influencing the concentration of neurotransmitters, such as GABA and glutamate, directly in the CNS [145]. In a recent randomized trial on people with AD, the daily administration of a probiotic mixture of *Lactobacillus acidophilus*, *Lactobacillus casei*, *Bifidobacterium bifidum*, and *Lactobacillus fermentum* for 12 weeks determined a statistically significant improvement in the mini-mental state exam (MMSE) score compared to controls. Positive changes in plasma metabolic markers were also reported [146]. Again, another recent meta-analysis reported positive effects on the cognitive function of probiotic administration in people with AD [147]. However, another recent study reported contrasting results. The authors did not observe any effect of a 12-week probiotic treatment in people with AD in terms of either cognitive functions or inflammatory markers [148].

Probiotics also showed promising results in people with PD. Recent studies have confirmed a possible positive role of the probiotic mixture in relieving constipation and motor symptoms [149,150,151]. In addition, even though the exact mechanism of action is unknown, probiotic treatment has been shown to modulate intestinal permeability, mucosal inflammation, and stimulate the production of SCFAs [152].

Finally, in a study on ALS patients, the *Rikenellaceae* family, belonging to *Bacteroidales* phylum, significantly increased after 6 months of probiotic administration [153].

### 8.3. Diet

Diet is a rapid and direct way to modify the gut microbiome composition and function. With diet alteration, the gut microbiome composition and the abundance of SCFAs and gut metabolites can change drastically [154]. Diet can influence inflammation inside the CNS through the GBA, and many different types of diets have been proposed as beneficial in preventing or ameliorating neurodegenerative diseases [154,155]. Among them, the Mediterranean diet is a well-known healthy diet rich in vegetables, whole grains, low in dairy, with olive oil as the main source of fat [156]. Recent studies reported how adherence to a Mediterranean diet can improve motor and cognitive symptoms in PD [157]. Moreover, a Mediterranean diet seems to reduce the risk of PD [158]. The Mediterranean diet has also shown beneficial effects on other forms of neurodegeneration, such as AD. Many, but not all, epidemiological studies confirmed a protective effect of the Mediterranean diet on neurodegeneration [101]. However, only a few large randomized clinical trials have been conducted so far, often with contrasting results [101].

A ketogenic diet is a nutritional program rich in fats and low in carbohydrates, developed in the 1900s as a treatment for epilepsy [159]. Recently, a ketogenic regimen has been investigated in neurodegenerative diseases, such as AD and PD, with promising results [160,161,162,163].

Lastly, as has been extensively reviewed elsewhere, a calorie restriction (CR) regimen has been shown to have beneficial effects on both neuroinflammation and neurodegeneration [157].

## 9. Conclusions

Studies in human and animal models of neurodegenerative diseases showed how the GBA might be involved in disease pathogenesis and a target for possible therapeutic approaches. The evidence linking the gut microbiome to neurodegeneration is compelling. However, studies on possible therapeutic manipulations of the gut microbiome are scarce and lack consistency. Despite being promising, this field is in its infancy, and more randomized clinical trials are needed to really understand its entire potential.

## Figures and Tables

**Table 1:** Human studies on the gut microbiome in neurodegenerative diseases. AD: Alzheimer’s disease, PD: Parkinson’s disease; ALS: Amyotrophic lateral sclerosis; HCs: Healthy controls; MCI: Mild cognitive impairment.

	Study Design	Analysis	Results	Ref
**AD**				
	Case control (40 Amyloid^+^, 33 Amyloid^3−^ subjects and 10 HCs)	Microbial DNA qPCR Assay Kit	Amyloid^+^ subjects: ↓ *E. rectale* and ↑ *Escherichia/Shigella* comapred to Amyloid^−^ and HCs	[16]
	Case control (25 AD and 25 HCs)	16S rRNA sequencing	AD: ↓ *Firmicutes* and *Actinobacteria* and ↑ *Bacteroides*	[17]
	Case control (24 AD, 33 other dementia, 51 HCs)	Shotgun metagenomic sequencing	AD: ↑ *Bacteroides* spp., *Alistipes* spp., *Odoribacter* spp., *Barnesiella* spp.; ↓ *Lachnoclostridium* spp.compared to HCs	[18]
	Case-control (33 AD, 32 aMCI and 32 HCs)	16S rRNA sequencing	AD: ↓ *Firmicutes*, increased *Proteobacteria* compared to HCs aMCI: ↑ *Bacteroides* compared to AD	[19]
	Case control (100 AD, 71 HCs)	16S rRNA sequencing	AD: ↓ *Faecalibacterium*, *Roseburia, Clostridium sensu stricto, Gemmiger, Dialister, Romboutsia, Coprococcus*, and *Butyricicoccus*	[20]
	Case-control (43 AD and 43 HCs)	16S rRNA sequencing	AD: ↓ in *Bacteroides* and increase in *Actinobacteria*	[21]
	Case-control (18 AD, 20 MCI, 18 HCs)	16S rRNA sequencing	AD: ↑ *Prevotella* and ↓ *Bacteroides, Lachnospira* compare to HCs MCI: ↑ *Prevotella* compared to HCs	[22]
**PD**				
	Case-control (51 PD, 48 HCs)	16S rRNA sequencing	PD: ↑ *Akkermansia* and *Prevotella*; ↓ *Lactobacillus*	[23]
	Case-control (193 PD, 22 PSP, 22 MSA and 113 HCs)	16S rRNA sequencing	PD: ↑ *Akkermansia* compared to HCs	[24]
	Case-control (76 PD, 76 HCs)	16S rRNA sequencing	PD: ↓ *Prevotella* and *Clostridium XIV*	[25]
	Case control (38 PD, 34 HCs)	16S rRNA sequencing	PD: ↑ *Roseburia, Blautia* and *Coprococcus*, ↓ *Faecalibacterium*	[26]
	Case-control (72 PD, 73 HCs)	16S rRNA sequencing	PD: ↑ *Prevotellaceae*	[27]
	Case-control (34 PD, 34 HCs)	16S rRNA sequencing	PD: ↓ *Bacteroidetes* and *Prevotellaceae*, ↑ *Enterobacteriaceae*	[28]
	Case-control (31 PD, 28 HCs)	Metagenomic shotgun sequencing	PD: ↑ *Akkermansia muciniphila*, ↓ *Prevotella copri* and *Eubacterium biforme*	[29]
	Case-control (197 PD, 130 HCs)	16S rRNA sequencing	PD: ↑ *Actinobacteria*, *Bacteroidetes* and *Firimicutes*	[30]
	Case-control (29 PD, 29 HCs)	16S rRNA sequencing	PD: ↑ *Lactobacillaceae*, *Bernesiellaceae* and *Enterococcaceae*	[31]
	Case-control (76 PD, 78 HCs)	16S and 18S rRNA sequencing	PD: ↑ *Akkermansia*	[32]
	Case-control (75 PD, 45 HCs)	16S rRNA sequencing	PD: ↓ *Lachnospiraceae*, increased *Bifidobacteriaceae*	[33]
	Case-control (80 PD, 72 HCs)	16S rRNA sequencing	PD: ↑ *Lactobacillaceae, Enterococcaceae* and *Enterobacteriaceae*; ↓ *Lachnospiraceae*	[34]
**ALS**				
	Case-control (6 ALS, 5 HCs)	16S rRNA sequencing	ALS: ↓ *Bacteroides, Prevotella* and *Escherichia*; increased *Faecalibacterium, Anaerosipes* and *Lachnospira*	[35]
	Case-control (25 ALS, 32 HCs)	16S rRNA sequencing	No significant difference	[36]
	Case-control (20 ALS, 20 HCs)	16S rRNA sequencing	ALS: ↑ *Bacterodetes*, ↓ *Firmicutes*	[37]
	Case-control (66 ALS, 61 HCs)	Metagenomic shotgun sequencing	ALS: ↓ *Eubacterium rectale* and *Roseburia intestinalis*	[38]
	Case-control (10 ALS, 10 HCs)	16S rRNA sequencing	ALS: ↓ *Prevotella*	[39]

## Data Availability

Not applicable.
